# Cellular Heterogeneity of Mesenchymal Stem/Stromal Cells in the Bone Marrow

**DOI:** 10.3389/fcell.2021.689366

**Published:** 2021-07-06

**Authors:** Yo Mabuchi, Chikako Okawara, Simón Méndez-Ferrer, Chihiro Akazawa

**Affiliations:** ^1^Wellcome-MRC Cambridge Stem Cell Institute, Department of Hematology, NHS Blood and Transplant, University of Cambridge, Cambridge, United Kingdom; ^2^Department of Biochemistry and Biophysics, Graduate School of Medical and Dental Sciences, Tokyo Medical and Dental University, Tokyo, Japan; ^3^Development of Innovation in Fundamental and Scientific Nursing Care, Graduate School of Health Care Sciences, Tokyo Medical and Dental University, Tokyo, Japan; ^4^Intractable Disease Research Centre, Juntendo University School of Medicine, Tokyo, Japan

**Keywords:** mesenchymal stem cells, heterogeneity, stem cell characterization, cell surface marker, myeloproliferative neoplasm, single-cell analysis

## Abstract

Mesenchymal stem/stromal cells (MSCs) are present in various body tissues and help in maintaining homeostasis. The stemness of MSCs has been evaluated *in vitro*. In addition, analyses of cell surface antigens and gene expression patterns have shown that MSCs comprise a heterogeneous population, and the diverse and complex nature of MSCs makes it difficult to identify the specific roles in diseases. There is a lack of understanding regarding the classification of MSC properties. In this review, we explore the characteristics of heterogeneous MSC populations based on their markers and gene expression profiles. We integrated the contents of previously reported single-cell analysis data to better understand the properties of mesenchymal cell populations. In addition, the cell populations involved in the development of myeloproliferative neoplasms (MPNs) are outlined. Owing to the diversity of terms used to describe MSCs, we used the text mining technology to extract topics from MSC research articles. Recent advances in technology could improve our understanding of the diversity of MSCs and help us evaluate cell populations.

## Introduction

Tissue formation during vertebrate development is a spatiotemporally dynamic process. Skeletal stem cells (SSCs) and mesenchymal stem/stromal cells (MSCs) are involved in tissue formation (Bianco et al., 2006, 2013; Chan et al., 2015; Worthley et al., 2015; Kurenkova et al., 2020). The MSCs present in adults are derived primarily from the mesoderm and partly from the neural crest (Takashima et al., 2007; Nagoshi et al., 2008; Isern et al., 2014). MSCs were first identified by Freidenstein and colleagues who reported that cells in the BM were capable of transforming into bone tissue (Friedenstein et al., 1968, 1974); they were subsequently isolated from somatic tissues, including dental pulp, synovium tissue, and adipose tissue (Ogata et al., 2015; Yasui et al., 2016; Suto et al., 2020). MSCs in adult bone marrow (BM) are defined as cells with the ability to differentiate into cells of mesenchymal lineage and can be cultured using serum-containing media. The essential features of the MSC populations have been defined by the International Society for Cell Therapy as follows: (1) adhesion to plastic surfaces under culture conditions, (2) expression of cell surface markers CD44, CD90, CD105, and CD73, (3) lack of expression of hematopoietic markers, and (4) the ability to differentiate into osteoblasts, chondroblasts, and adipocytes (Dominici et al., 2006). The presence of MSCs was confirmed in the human BM and their potential application in medical treatments was suggested owing to their pluripotency and ability to readily proliferate *in vitro* (Pittenger et al., 1999; Bianco et al., 2008; Crisan et al., 2008). Although these cells can differentiate into cells of the mesenchymal lineage *in vitro*, there is insufficient evidence as to whether they can differentiate under physiological conditions (Sacchetti et al., 2016). Prolonged culture of MSCs *in vitro* leads to a significant loss of the differentiation and proliferation potentials (Kim et al., 2009).

Research on MSCs thus far can be roughly divided into three stages. The first stage involved the discovery of MSCs and the analysis of their multi-lineage potential (1970 onward), the second constitutes functional analysis, which remains a subject of investigation, using transplantation in mouse models (from 2000 onward), and the medical application of MSCs represents the third stage. MSC transplantation therapy has been used as a substitute for the long-term transplantation of mesenchymal tissue (Bianco et al., 2013). The next stage of research on MSCs could establish them as mediators of inflammation and as source of transiently expressed secretory factors (cytokines and exosomes), a phenomenon that could have various applications (El Agha et al., 2017). MSCs have been used in regenerative medicine; however, the mechanism of action of these cells remains to be determined. The underlying limitation is that the MSC population is heterogeneous. Furthermore, MSCs have been isolated based on culture conditions and defined by cell surface antigens. Consequently, there is a lack of a common understanding regarding the high-resolution indicators of MSC properties in current research.

Previous studies have suggested that the expression of cell surface markers may alter under certain culture conditions (Stagg et al., 2006). In other words, *in vitro* culture and the identification of cell surface antigens cannot serve as sole guarantors of stemness. To define a cell as a stem cell, it must meet functional criteria in addition to presenting a specific cell surface antigen profile (Nolta et al., 2020). The information gained from single-cell technology elucidates the stem cell property and establishes new criteria for identification (Tanay and Regev, 2017; Stuart and Satija, 2019). This review focuses on the heterogeneity of mouse BM-MSCs. In addition, we discuss the pathophysiology of MSC-related diseases, which have been actively studied in recent years.

## Dissecting MSCs Using Cell Surface Markers and Transcriptional Profiles

Hematopoietic stem cells (HSCs) have been identified using purification techniques that target cell surface antigens (Positive markers for Sca-1, c-Kit, and CD150; Negative markers for Lineage, CD34, CD48, and CD135) (Osawa et al., 1996; Notta et al., 2011; Doulatov et al., 2012). MSCs have been reported in the non-hematopoietic fraction, both human and mouse MSCs have been identified based on the negative expression of surface markers (leukocyte common antigen: CD45, platelet endothelial cell adhesion molecule: CD31, and erythroid cell marker). Some of the positive markers used are common to humans and mice (CD73, CD90, and CD105) (Mabuchi et al., 2013a). However, the specific positive marker used tend to be different for each species [for human: CD146, and CD271, for mouse: CD140a, Sca-1, and CD295 (leptin receptor)] (Sacchetti et al., 2007; Morikawa et al., 2009; Mabuchi et al., 2013b; Zhou et al., 2014). Although it is possible to concentrate MSCs with cell surface antigens, the purified MSC population may be functionally heterogeneous (proliferative and differentiating capacity) (Mabuchi et al., 2013b; Rennerfeldt and Van Vliet, 2016; Costa et al., 2021). MSCs are present in extramedullary sites and the gene expression profile and differentiation ability of these cells have been reported to differ depending on the tissue of origin (Ogata et al., 2015). The current stem cell definition criteria require the demonstration of self-renewal ability *in vivo*, which is usually tested through transplantation procedures (Sacchetti et al., 2007; Mendez-Ferrer et al., 2010). Donor stem cells are tracked using genetic markers such as the Y chromosome and expression reporters such as GFP and lacZ. For accurate analysis of stem cell capacity, it is important to prospectively purify the donor cells and assess their clonality. In addition, it is necessary to evaluate the presence of transplanted cells as well as the *in vivo* functions of these cells. To understand the plasticity and biological role of MSCs, lineage tracing of donor cells is essential. However, an unresolved issue after MSC transplantation is their inefficient and transient engraftment; hence, the true function and properties of these cells remain unclear.

Cellular heterogeneity is a universally recognized feature of living tissues and cells, including MSCs (Rennerfeldt and Van Vliet, 2016; Costa et al., 2021). Even in nearly pure cell populations, gene expression profile can vary in individual cells due to variation in intrinsic regulatory systems and the extrinsic microenvironment. Human MSCs isolated under criteria established by The International Society for Cell Therapy are difficult to distinguish from similar cell populations and are not well characterized. Transcriptomics could be used to determine well-defined MSC gene signature. RNA-Seq results from MSC primary cultured cells from human BM and placenta were consistent with standard MSC marker expression levels (Roson-Burgo et al., 2014). Conversely, when the expression of CD146, nestin, and CD271 was confirmed in placenta-derived cells, the expression of only CD146 and nestin was detected (Roson-Burgo et al., 2014). In addition, integration of several public datasets (microarray and RNA-Seq datasets) demonstrated that tissue-specific gene expression patterns of adipose tissue, chorionic placenta, BM mesenchymal stem cells, and cutaneous fibroblasts can be obtained (Roson-Burgo et al., 2016). MCAM (CD146) is a common marker in this subtype of MSCs (Sacchetti et al., 2007). Gene expression in BM-MSCs, HSCs, lymphocytes, fibroblasts, and osteoblasts has been used to create regulatory gene networks (Roson-Burgo et al., 2016). These algorithms were used to identify potential master regulators of genes that are upregulated in BM-MSCs and genes that exhibit hypomethylation (EPAS1, NFE2L1, SNAI2, STAB2, TEAD1, and TULP3) (Sanchez-Luis et al., 2020). Furthermore, the gene encoding Frizzled 5, i.e., *FZD5* is highly expressed in undifferentiated human MSCs, indicating that not only canonical but also non-canonical Wnt signaling is important for maintaining stemness (Harada et al., 2020). These findings may be used as a functional indicator of MSCs.

## Biological Function of the Hematopoietic Niche and Stromal Cells

A stem cell niche is defined as the microenvironment where stem cells reside and receive stimuli that determine their fate. The niche of HSCs was first proposed by Schofield (1978) and the existence of various other niches that maintain stem cells throughout life has since been reported (Yamazaki et al., 2011; Ding et al., 2012). Like other adult stem cells, MSCs maintain their capacity for self-renewal and differentiation into various cell types, while maintaining a relatively steady stem cell pool during their lifespan (Mendez-Ferrer et al., 2010; Kfoury and Scadden, 2015). The stem-cell niche provides a physical location that supports stem cells (closed niche). However, some tissues harbor less physically constrained niches that provide a more complex system for supporting stem cells (open niche) (Watt and Hogan, 2000; Yoshida, 2018). Spermatogenic stem cells are interspersed among differentiating progeny while undergoing self-renewal and differentiation. Some spermatogenic stem cells proliferate and other spermatogenic stem cells exit the stem cell compartment after differentiation (population asymmetry). In an “open niche” microenvironment, self-renewal and differentiation are perfectly balanced at the population level (Hara et al., 2014). The BM HSC niche is in line with the closed niche concept, although it contains a variety of cell types including osteoprogenitors, fibroblastic reticular cells and endothelial cells, and it is directly influenced by other cell types, such as macrophages, megakaryocytes, Treg cells, nerve fibers, and associated Schwann cells (Crane et al., 2017).

Cells with varied potential exist in the adult BM; hence, the purification method can result in differences in apparent stem cell capacity, which can lead to different results. For example, some stem cell markers (such as CD34) are expressed in a dynamic pattern and are associated with the activation state of the stem cells (Sato et al., 1999). Stem cell phylogenetic tracking is necessary to distinguish between the heterogeneity of the stem cell population and the plasticity of the cellular response *in vivo*. Moreover, the diversity of stem cell types in the BM should be considered in studies based on cultured MSCs. Clinical therapies require large numbers of MSCs and cell growth *in vitro.* However, long-term *in vitro* culture can negatively affect stem cell capabilities (Turinetto et al., 2016). Reports have shown that the stiffness of the hydrogel in which MSCs are encased for experiments can affect cytokine secretion and immunomodulatory marker expression in MSCs (Darnell et al., 2018). Furthermore, the properties of MSCs vary with cell density, while the angiogenesis-promoting properties of MSCs may be lost in certain culture conditions (Ren et al., 2015). The BM microenvironment provides an environment that affects not only HSCs but also various other cells. The BM environment is rich in cytokines and growth factors and may be particularly important for maintaining the developmental potential and plasticity of these cells. This plasticity and cell heterogeneity is the result of biological defense that maintains a wide range of developmental and differentiation abilities in response to various disorders and stresses before their fate is determined. In adults, the broad capacity of these cells may be maintained by the microenvironment in closed tissues such as BM. Several reports have described morphological and functional changes in BM stromal cells due to disruption of the niche mechanisms in patients with various hematological diseases (Schepers et al., 2015). Hence, niche cells are diverse, and important for pathophysiology of blood diseases.

Mesenchymal-derived cells, which are widely distributed in the BM, have been reported as important players in the HSC niche. These cells, which include Cxcl12-rich reticular (CAR) cells and leptin receptor (LepR) expressing cells, have been shown to overlap with cells marked with GFP under the regulatory element of the nestin promoter (Nes-GFP^+^) (Sugiyama et al., 2006; Mendez-Ferrer et al., 2010; Ding et al., 2012). Whole-mount image analysis showed that stromal cells with a bright GFP signal in Nes-GFP^+^ cells were associated with BM arterioles (NG2 positive). Meanwhile, Nes-GFP^+^ cells with low GFP levels were distributed in sinusoidal capillaries (LepR-Cre positive) (Kunisaki et al., 2013). Deletion of Scf or Cxcl12 from Nes-GFP^+^ cells depleted BM-HSCs. Conditional removal of Scf from LepR-Cre/tdTomato^+^ cells reduced the number of BM HSCs (Ding et al., 2012; Oguro et al., 2013). Selective deletion of Cxcl12 from NG2-Cre/tdTomato^+^ cells caused the HSCs to decrease and altered HSC localization in BM (Asada et al., 2017). Regarding the effect of HSCs on peripheral mobilization, LepR-Cre/tdTomato^+^ cells were induced by the deletion of Cxcl12 (Ding and Morrison, 2013). By classifying Nes-GFP^+^ cells into clusters, it became possible to prove the contribution of different cytokines in BM-niche cells (Ding and Morrison, 2013; Asada et al., 2017). LepR^+^ cells are a major source of bone and adipocytes in the adult BM and proliferate and are able to produce mesenchymal lineage cells in response to injury and transplant (Zhou et al., 2014; Yue et al., 2016). Osterix (Osx), a marker of mature osteocytes, is not expressed in Nestin-GFP or LepR^+^ cells, however, neonatal Osx-Cre-ERT2-labeled cells are precursors of LepR^+^ and Nestin-GFP cells in the adult BM (Mizoguchi et al., 2014). In the adult BM, after radiation or chemotherapy, adipocytes become abundant, while LepR^+^ cells are reduced (Zhou et al., 2014). Adipoq-Cre/ER^+^ progenitor cells proliferate after irradiation and generate BM adipocytes that secrete Scf (Zhou et al., 2017). The effects of niche cell depletion on various hematopoietic cells need to be analyzed, including the association between the influencing cell types and their offspring; experiments using reporter mice are limited. Comprehensive niche cell analysis using an unbiased analysis method is considered important.

## Characterization of Non-Hematopoietic Cells at Single-Cell Resolution

The scRNA-seq is a powerful tool for characterizing such heterogeneous cell populations (Tanay and Regev, 2017; Stuart and Satija, 2019), allowing rapid analysis of MSC diversity. Previous reports identified mouse SSCs (mSSCs: CD45^–^Ter119^–^Tie2^–^AlphaV^+^Thy^–^6C3^–^CD105^–^CD200^+^) have differentiation potential similar to that of MSCs (Chan et al., 2015). The authors used scRNA-seq to compare human SSCs (hSSCs: PDPN^+^CD146^–^CD73^+^CD164^+^) from fetal BM, adult BM, adipose stroma, and iPSC-derived cells. This showed that adult hSSCs were heterogeneous compared to fetal, adipose stroma-derived, or iPSC-derived hSSCs. It was suggested that single-cell analysis results yield dissimilar profiles, even though they are functionally similar in terms of differentiation ability (Chan et al., 2018). Previous reports have shown that the BM stroma fraction has different cellular functions in Nes-GFP^bright^ (NG2^+^), Nes-GFP^dim^ (LepR^+^), and Nestin negative cells (Mendez-Ferrer et al., 2010; Kunisaki et al., 2013). High-resolution classification of MSC populations using scRNA-seq and attempts to classify the niche cells surrounding HSCs are ongoing as previously reported (Table 1; Baryawno et al., 2019; Tikhonova et al., 2019; Wolock et al., 2019; Baccin et al., 2020; Leimkuhler et al., 2020; Zhong et al., 2020). Moreover, crosstalk between cancer cells and mesenchymal populations impairs normal tissue function, leading to cancer development (Baryawno et al., 2019). scRNA-seq from mouse stromal cells has shown that the development of AML impairs mesenchymal cells-induced bone formation and differentiation, and suppresses the regulatory molecules required for normal hematopoiesis (Baryawno et al., 2019). The transcriptional landscapes of vascular, perivascular, and osteoblast cell populations of mouse BM during homeostasis and under stress-induced conditions have also been mapped using scRNA-seq (Tikhonova et al., 2019). Vascular Notch delta-like ligand, which is encoded by Dll1 and Dll4, is downregulated after intraperitoneal administration of fluorouracil (Tikhonova et al., 2019). These MSC populations represented by Nestin and LepR have similar gene expression (Cxcl12, Kitl, Angpt1, and Spp1) (Mendez-Ferrer et al., 2010; Kunisaki et al., 2013; Baryawno et al., 2019; Tikhonova et al., 2019). These populations can be further sub-divided into 3–4 subsets using Nestin, Grem1, Angpt1, and Spp1 gene expressions (Baryawno et al., 2019; Tikhonova et al., 2019). The expression of key genes indicating MSC gene expression (Cxcl12, Kitl) is also common to scRNA-seq data from other researchers (Wolock et al., 2019; Baccin et al., 2020; Leimkuhler et al., 2020). Molecules identified as MSC surface markers (Sca-1, Pdgfra, and Thy-1) were also specifically expressed (Baccin et al., 2020; Leimkuhler et al., 2020; Zhong et al., 2020).

**TABLE 1:** Classification of mouse BM cell population.

Cell type	Name	Specific genes	Population	Spices	Analysis sample	References
MSC	Nestin-MSC	Nestin, Cxcl12, Kitl, Angpt1, Vcam1, Spp1	3 subsets	Mouse	CD45 (-)	Mendez-Ferrer et al., 2010 Nature Kunisaki et al., 2013 Nature
MSC	Lepr-MSC	Lepr, Cxcl12, Kitl, Angpt1, Adipoq	4 subsets	Mouse	Lineage (-) CD71 (-) Ter119 (-)	Baryawno et al., 2019 Cell
MSC	LEPR +	Lepr, Mgp, Lpl, Wif1, Spp1, Ibsp1	4 subsets	Mouse	lineage-specific Cre-transgenic mice	Tikhonova et al., 2019 Nature
MSC	Mesenchymal stromal cells, Pre-adipocyte/Adipocyte	Cxcl12, Kitl, Spp1, Adipoq,	3 subset	Mouse	CD45 (-) CD31 (-) Ter119 (-)	Wolock et al., 2019 Cell Reports
MSC	Mesenchymal progenitors (Early)	Ly6a (Sca-1), Thy1, Mfap5, Clec3b	2 subsets	Mouse	Col2- lineage-specific Cre-transgenic mice	Zhong et al., 2020 eLife
MSC	Mesenchymal stromal cells	Pdgfra, Lepr, Cxcl12, Kitl, Pdgfrb	4 subsets	Mouse	BM fibrosis model	Leimkuhler et al., 2020 Cell Stem Cell
MSC	Adipo-CAR	Cxcl12, Kitl, Pdgfra, Vcam1	2 subsets	Mouse		Baccin et al., 2020 Nat Cell Biol
Progenitor	Pre-osteo/chondrocyte progenitor	Postn, Wif1, Mmp9, Kcnk2, Limch	2 subsets	Mouse	CD45 (-) CD31 (-) Ter119 (-)	Wolock et al., 2019 Cell Reports
Progenitor	Mesenchymal progenitors (Late)	Tnn, Postn, Ostn, Dkk3	1 subset	Mouse	Col2- lineage-specific Cre-transgenic mice	Zhong et al., 2020 eLife
Progenitor	Adipocyte progenitor	Cebpa, Pparg, Lpl, Adipoq, Apoe	1 subset	Mouse	Col2- lineage-specific Cre-transgenic mice	Zhong et al., 2020 eLife
Osteo-lineage cells	Osteolineage cells	Runx2, Sp7, Bglap	2 subsets	Mouse	Lineage (-) CD71 (-) Ter119 (-)	Baryawno et al., 2019 Cell
Osteo-lineage cells	COL2.3+	Col1a1, Col16a1, Fbn1, Bglap, Tnn	3 subsets	Mouse	lineage-specific Cre-transgenic mice	Tikhonova et al., 2019 Nature
Osteo-lineage cells	OSTEO-CAR, Osteoblasts, Ng^2 +^MSCs	Bglap, Sp7, Spp1	3 subsets	Mouse		Baccin et al., 2020 Nat Cell Biol.
Osteo-lineage cells	Pro-osteoblast	Col1a1, Bglap, Col11a2, Col11a1, Bglap2	1 subset	Mouse	CD45 (-) CD31 (-) Ter119 (-)	Wolock et al., 2019 Cell Reports
Osteo-lineage cells	Osteoblasts/Osteocyte	Sp7, Runx2, Col1a1, Ibsp, Bglap2, Dmp1	2 subsets	Mouse	Col2- lineage-specific Cre-transgenic mice	Zhong et al., 2020 eLife
Osteo-lineage cells	Osteolineage cells	Sp7, Bglap, Bglap2, Alpl	1 subset	Mouse	BM fibrosis model	Leimkuhler et al., 2020 Cell Stem Cell
Chondro-lineage cells	Chondrocytes	Sox9, Col11a2, Acan, Col2a1	5 subsets	Mouse	Lineage (-) CD71 (-) Ter119 (-)	Baryawno et al., 2019 Cell
Chondro-lineage cells	Pro-chondrocyte	Dmp1, Ackr3, Spp1, Ank, Cd44	1 subset	Mouse	CD45 (-) CD31 (-) Ter119 (-)	Wolock et al., 2019 Cell Reports
Chondro-lineage cells	Chondrocyte	Sox9, Col2a1, Acan, Ihh	2 subsets	Mouse	Col2- lineage-specific Cre-transgenic mice	Zhong et al., 2020 eLife
Chondro-lineage cells	Chondrocyte	Sox9, Acan	1 subset	Mouse		Baccin et al., 2020 Nat Cell Biol
Fibroblasts	Fibroblasts	S100a4, Dcn, Sema3c	5 subsets	Mouse	Lineage (-) CD71 (-) Ter119 (-)	Baryawno et al., 2019 Cell
Fibroblasts	Fibroblasts	Col1a1, Ly6a (Sca-1), Dcn	4 subsets	Mouse		Baccin et al., 2020 Nat Cell Biol.
Fibroblasts	Arterial Fibroblasts	Cd34, Ly6a (Sca-1), Ly6c1, Sparcl1	4 subsets	Mouse	BM fibrosis model	Leimkuhler et al., 2020 Cell Stem Cell
Endothelial cells	Endothelial cells	Kdr, Cdh5, Pecam1	3 subsets	Mouse	Lineage (-) CD71 (-) Ter119 (-)	Baryawno et al., 2019 Cell
Endothelial cells	VE-Cad +	Cdh5, Ly6a (Sca-1), Stab2	2 subsets	Mouse	lineage-specific Cre-transgenic mice	Tikhonova et al., 2019 Nature
Endothelial cells	Endothelial cells	Cdh5, Emcn	2 subsets	Mouse		Baccin et al., 2020 Nat Cell Biol
Pericytes	Pericytes	Acta2, Myh11, Rgs5	3 subsets	Mouse	Lineage (-) CD71 (-) Ter119 (-)	Baryawno et al., 2019 Cell
Pericytes	Myofibroblasts	Acta2	1 subset	Mouse		Baccin et al., 2020 Nat Cell Biol
Schwann cells	Schwann cells	Mag, Mog	1 subset	Mouse		Baccin et al., 2020 Nat Cell Biol
Schwann cells	Schwann cell progenitors	Mog, Mal, Sox10, Mobp	2 subsets	Mouse	BM fibrosis model	Leimkuhler et al., 2020 Cell Stem Cell

Integration of scRNA seq data suggests that non-MSC fractions in the BM can also vary (Table 1). A “Progenitor” population has been identified to express Postn (Periostin) and adipocyte-related genes (Pparg, Lpl, and Adipoq) (Wolock et al., 2019; Zhong et al., 2020). Cell populations that are committed to differentiate into cells of the bone lineage are identified as “Osteo-lineage cells.” Bone-related genes (Col1a1, Bglap, and Sp7) are prominently expressed in the Osteo-lineage fraction (Baryawno et al., 2019; Tikhonova et al., 2019; Wolock et al., 2019; Baccin et al., 2020; Leimkuhler et al., 2020; Zhong et al., 2020). “Chondro-lineage cells” are characterized by expression of genes such as Sox9 and Acan (Baryawno et al., 2019; Wolock et al., 2019; Baccin et al., 2020; Zhong et al., 2020). The cell population labeled “Fibroblasts” is diverse and includes the above stem cells and cells that do not belong to lineage cells. The “Fibroblasts” population seems to contain more than four subsets (Baryawno et al., 2019; Baccin et al., 2020; Leimkuhler et al., 2020). Interestingly, even the endothelial cells present in the BM seem to have two cell population (Baryawno et al., 2019; Tikhonova et al., 2019; Baccin et al., 2020). The BM vasculature is composed of two main types of blood vessels, i.e., arterial blood vessels and sinusoids. These results are supported by previous findings that arterial endothelial cells exhibit an elongated nuclear morphology (express Sca-1 and nestin markers) and that sinusoid endothelial cells exhibit a rounded nuclear morphology (do not express Sca-1 and nestin) (Itkin et al., 2016). Heterogeneity has been reported in established cell populations such as “Pericytes” (Acta2) and “Schwann cells” (Mog) (Baryawno et al., 2019; Baccin et al., 2020; Leimkuhler et al., 2020).

Single-cell technology has made remarkable progress in recent years, e.g., Quartz-seq, Drop-seq, and RamDA-seq (RNA) (Sasagawa et al., 2013; Macosko et al., 2015; Hayashi et al., 2018), SCI-seq (genome sequence) (Vitak et al., 2017), scBS-seq (DNA methylation) (Smallwood et al., 2014), scATAC-seq (chromatin accessibility) (Buenrostro et al., 2015), scChip-seq (histone modification) (Gomez et al., 2013), and smFISH (spatial positioning) (Raj et al., 2008). Further, technologies have been established by coupling with scRNA-seq for providing higher-resolution information. There are single-cell methods that combine with surface antigens (REAP-seq and CITE-seq) (Peterson et al., 2017; Stoeckius et al., 2017) and lineage tracing technology (Alemany et al., 2018; Raj et al., 2018; Spanjaard et al., 2018). Single-cell protein analysis using mass spectrometry (CyTOF) has been used to analyze BM stromal mapping under homeostatic and stress conditions (Severe et al., 2019). Irradiation eliminated most of LepR^+^ and Nestin^+^ niche populations during HSC transplantation. The CD73^+^NGFR^high+^ population was retained and expressed high levels of niche cytokines (Severe et al., 2019). The expression mapping at single-cell protein revealed information regarding different sets of stromal cells in the BM and provided alterations upon exposure to stressful environments at high resolutions. In addition, single-cell atlases are available for tissues and organs, including adipose tissue (Sun et al., 2020), heart (Litvinukova et al., 2020), lungs (Travaglini et al., 2020), lymph nodes (Rodda et al., 2018), and hair follicles (Gupta et al., 2019). The, single-cell technology has revealed a larger subset of cells and specific marker genes, which may help explain the regulatory networks underlying physiological and pathological conditions (Buechler et al., 2021). These reports have improved our understanding of the identity of tissue-specific cell types and tissue diseases.

## Heterogeneity of Cells and Pathophysiology Involved in MPN

Myeloproliferative disorders (MPDs) are an example of phenotypic and functional MSC heterogeneity in disease, which include chronic myeloid leukemia (CML) and BCR-ABL-negative myeloproliferative neoplasms (MPNs), such as polycythemia vera (PV), essential thrombocythemia (ET), and primary myelofibrosis (PMF) (Arber et al., 2016). MPDs are genetic abnormalities in HSCs that result in the monoclonal proliferation of one or more types of blood cells, eventually leading to myelopoietic failure and myelofibrosis osteosclerosis. CML is caused by a chromosomal translocation, *t*(9;22), in HSCs that results in formation of the *BCR/ABL1* fusion gene. The shorter chromosome 22 formed by chromosome translocations is called the Philadelphia (Ph) chromosome. The ability of HSCs to differentiate is not inhibited in BM, and granulocytic cells are markedly increased in CML. PV is a genetic abnormality occurring at the level of HSCs that results in pancytoplasmic increase, especially in erythrocytes; more than 95% of PVs are associated with a mutation in the Janus kinase 2 (*JAK2*) gene. ET is a myeloproliferative tumor caused by a genetic abnormality in HSCs, resulting in abnormal proliferation of megakaryocytes (MKs), and significant increase in platelet numbers. Mutations in *JAK2* are found in approximately half of ET cases, with most of the other half carrying mutations in the calreticulin (*CALR*) gene. PMF is a myeloproliferative tumor in which myeloid cells, including MKs, proliferate due to a genetic abnormality that occurs at the level of HSCs. This disease is characterized by extensive fibrosis of the BM, splenomegaly owing to extramedullary hematopoiesis, and erythroblasts and myeloblasts in the peripheral blood. PMF patients typically test negative for the Ph *BCR/ABL1* gene; additionally, an associated mutation identified in *JAK2* is present in approximately 50% of MPF cases. However, 10% of PMF cases are considered triple negative, having none of the three common mutations (*JAK2*, *MPL*, or *CALR*) (Campbell and Green, 2006; Cazzola and Kralovics, 2014). In addition to age and sex, specific genomic mutations have been associated with the development of MPNs (Tabarroki and Tiu, 2014). MPN, a clonal disorder of HSCs and the ensuing fibrosis, is a secondary effect caused by the release of cytokines from progenitor cells (Jacobson et al., 1978). Abnormal production of growth factors (PDGF, FGF, VEGF, and TGF-β1) plays important roles the pathological changes associated with MPNs (Ciurea et al., 2007; Tomuleasa et al., 2018; Ozono et al., 2020). Excessive release of growth factors stimulates fibroblasts thereby inducing fibrosis and unbalanced osteoblast proliferation, resulting in osteosclerosis and neoangiogenesis (Chagraoui et al., 2003). Clonal HSC-derived MKs is an important source of fibrosis-related molecules (Schmitt et al., 2002; Zetterberg et al., 2014).

The BM microenvironment plays an active and important role in MPN development (Lataillade et al., 2008). Previous reports showed a model of MPN in mice driven by conditional and inducible expression of the human JAK2 (V617) under Mx1-Cre control was used. Nestin^+^ MSCs reductions were consistently confirmed in MPN patients and mouse MPN models. Interleukin-1b produced by mutant HSCs causes Schwann cell apoptosis, resulting in a decrease in Nestin^+^ MSCs that allows disease progression (Arranz et al., 2014). Gli1^+^ MSCs can become myofibroblasts that cause BM fibrosis. It was found to reduce the severity of fibrosis by inhibiting the proliferation of Gli1^+^ cells and the differentiation of myofibroblasts. Gli1 expression is significantly correlated with disease severity, as human BM fibrosis has similar activity (Schneider et al., 2017). It has been demonstrated that LepR^+^ MSCs proliferate and are involved in fibrosis in MPN model mice in which Tpo overexpressing BM cells are transplanted into irradiated mice. Activation of the PDGFRA pathway in LepR^+^ MSCs resulted in cell proliferation and extramedullary hematopoiesis characteristic of PMF (Decker et al., 2017). The crosstalk between HSCs and these MSC populations and their contributions to diseases remain to be elucidated (Sena et al., 2017).

In recent years, the Schneider group has succeeded in creating a comprehensive map of MPN stroma cells at the single-cell level (Leimkuhler et al., 2020). Comparison of cells before and after fibrosis showed that the disease stroma cells caused activation of TGF-β ligands and receptors, leading to abnormal cell proliferation and increased extracellular matrix secretion (Leimkuhler et al., 2020). The expression of S100A8/S100A9 in stroma cell cluster showed significant increase in patient plasma and mesenchymal tissue in the fibrotic phase of mouse models of MPN. The upregulated S100A8/S100A9 expression can be a stress response, which impairs normal hematopoiesis, and induces genotoxic stress (Schneider et al., 2016). Nestin^+^ MSC has been shown to support AML survival and chemotherapy recurrence through antioxidant defense mechanisms (Forte et al., 2020). The glutathione-dependent antioxidant pathways emerge as key players in the crosstalk between BM-MSCs and leukemia stem cells. Thus, the analysis of MSC diversity and changes in their properties can elucidate the identification of the cell population involved in disease and generate new candidate therapies.

## Diversity and Comprehensive Understanding of MSC Research

**FIGURE 1:**
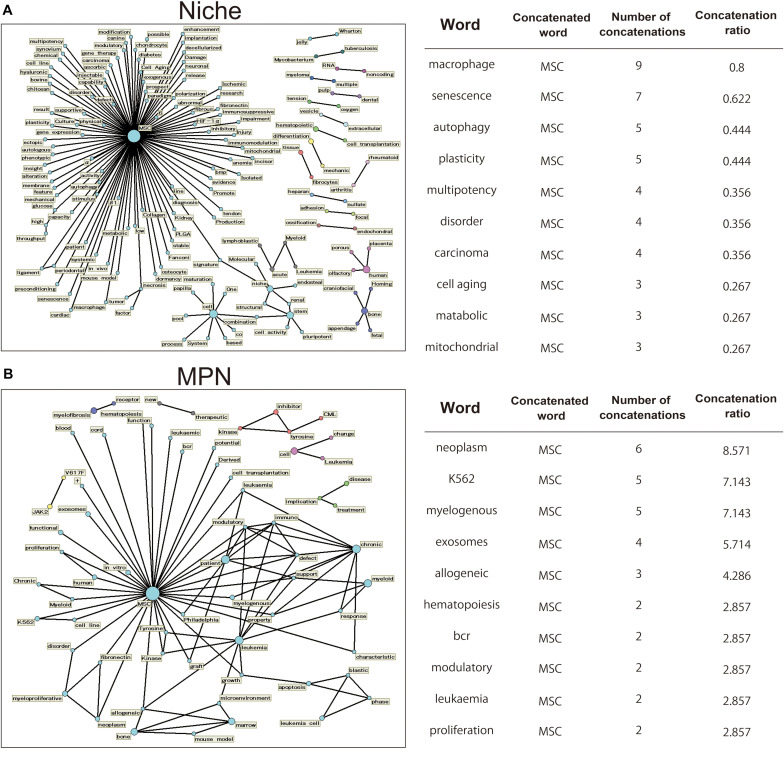
Analysis of word highly related to MSCs using the text mining method. Using PubMed, we extracted articles referring to MSCs in their titles using a collection of MeSH word that describe MSCs. We focused on the word related to “niche” or “MPN,” and created a term network using Text Mining Studio 6.3 (NTT DATA Mathematical Systems Inc.) with the following analysis conditions: “niche” and “MPN,” frequency > 2, confidence level 30%. **(A,B)** The obtained data are shown as a network diagram demonstrating the relations between words. Word-relationship diagram related to the word “niche” [**(A)** left] and “MPN” [**(B)** left]. Representative 10 words are shown on the right table [**(A)** right and **(B)** right]. “Concatenated word” is those concatenated with the representative word. “Number of concatenations” indicates the number of occurrences of concatenated words. “Concatenation ratio” indicates the ratio of concatenated articles in the extracted papers.

Different terms, criteria, and acronyms have been used to refer to MSCs. This diverse nomenclature includes terminology such as mesenchymal stem cells, mesenchymal stromal cells, and BM stromal cells (Mabuchi and Matsuzaki, 2016; Costa et al., 2021). Thus, the diversity of these words is a barrier to collecting a wide range of information about MSCs from the database. The text mining assesses the frequency of appearances of specific words and phrases, their co-occurrences, and time-series data, and may be a useful technique for analyzing trends in scientific literature without subjective bias (Watanabe et al., 2018; Lee et al., 2019). Therefore, we searched articles in the PubMed database and selected those related to MSCs. To ensure a comprehensive selection of articles on the topic of MSCs, we used Medical Subject Heading terms to collect scientific articles (30,849 articles, 1995–2020). Among them, we extracted a list of papers containing the following two keywords [“Niche”: 1,125 articles, “myeloproliferative neoplasms (MPN)”: 70 articles]. Then, words with high probability of occurring together in the title were extracted and their relationships are displayed in a network diagram (Figure 1). words that are closely related to each other are displayed connected by lines. The 147 related words were extracted for the titles of the treatises related to “niche” (Figure 1A, left). The number of clusters were directly associated with the MSC, consisted of the words “multipotency” and “plasticity” brought about by Niche, while “endosteal,” “stem,” and “pluripotent.” As characteristic clusters, clusters related to blood cancer called “Leulemia” and “myeloma” were also confirmed. Representative words are shown on the right table and Figure 1A (right). These results enrich the words that involved MSC function in niche. The 106 related words were extracted for the titles of the treatises related to “MPN” (Figure 1B, left). In clusters containing “MSC,” words related to diseases such as “leukemia” were mainly displayed. In addition, clusters associated with MPN such as “JAK2/V617F” and “CML” could be confirmed. These results indicate that there is an intimate relationship between MPNs and MSCs (Figure 1B, right).

## Discussion

This review summarized the diversity of MSCs and literature based on single cell analysis, and outlined what cell populations exist and how they are linked to cell therapy candidates. Diversity of MSCs and how stem cells balance their self-renewal and differentiation abilities are important themes in biological and medical research. However, the heterogeneity and plasticity of these cells—probably evolved during ontogeny as a mechanism to fulfill different functions while retaining their potential for self-renewal—pose significant challenges. Furthermore, it is currently considered that tissue stem cells move between two or more metastable states (self-renewal bias and differentiation bias) (Graf and Stadtfeld, 2008). The oscillations between these subsets involve changes in histone modifications, which are expected to help complement the function of MSCs in response to environmental changes. Therefore, further research is needed to address the functional heterogeneity of MSCs in health and disease. However, as the level of information and resolution of scRNA-seq analysis increases, there are limitations that make integrated analysis difficult. An additional limitation to current single-cell RNA-seq analyses is the low sequencing depth, which may capture only highly expressed mRNAs. In addition, the lack of localization information regarding the cluster cell population is also a problem to be solved in the future. Additional data could help us better understand the regulatory functions of MSCs in different tissues and could assist in more efficient treatment of diseases where MSCs play a pathophysiological role, such as MPNs.

## Author Contributions

YM and SM-F: conception and design of the study, data analysis and interpretation, manuscript writing, and final approval of the manuscript. CO: collection and assembly of data and final approval of the manuscript. CA: data analysis and interpretation, manuscript writing, and final approval of the manuscript. All authors contributed to the article and approved the submitted version.

## Conflict of Interest

The authors declare that the research was conducted in the absence of any commercial or financial relationships that could be construed as a potential conflict of interest.

## References

[B1] Alemany A. Florescu M. Baron C. S. Peterson-Maduro J. van Oudenaarden A. (2018). Whole-organism clone tracing using single-cell sequencing. *Nature* 556 108–112. 10.1038/nature25969 29590089

[B2] Arber D. A. Orazi A. Hasserjian R. Thiele J. Borowitz M. J. Le Beau M. M. (2016). The 2016 revision to the World Health Organization classification of myeloid neoplasms and acute leukemia. *Blood* 127 2391–2405. 10.1182/blood-2016-03-643544 27069254

[B3] Arranz L. Sanchez-Aguilera A. Martin-Perez D. Isern J. Langa X. Tzankov A. (2014). Neuropathy of haematopoietic stem cell niche is essential for myeloproliferative neoplasms. *Nature* 512 78–81. 10.1038/nature13383 25043017

[B4] Asada N. Kunisaki Y. Pierce H. Wang Z. Fernandez N. F. Birbrair A. (2017). Differential cytokine contributions of perivascular haematopoietic stem cell niches. *Nat. Cell Biol.* 19 214–223. 10.1038/ncb3475 28218906 PMC5467892

[B5] Baccin C. Al-Sabah J. Velten L. Helbling P. M. Grunschlager F. Hernandez-Malmierca P. (2020). Combined single-cell and spatial transcriptomics reveal the molecular, cellular and spatial bone marrow niche organization. *Nat. Cell Biol.* 22 38–48. 10.1038/s41556-019-0439-6 31871321 PMC7610809

[B6] Baryawno N. Przybylski D. Kowalczyk M. S. Kfoury Y. Severe N. Gustafsson K. (2019). A cellular taxonomy of the bone marrow stroma in homeostasis and leukemia. *Cell* 177 1915–1932.e16. 10.1016/j.cell.2019.04.040 31130381 PMC6570562

[B7] Bianco P. Cao X. Frenette P. S. Mao J. J. Robey P. G. Simmons P. J. (2013). The meaning, the sense and the significance: translating the science of mesenchymal stem cells into medicine. *Nat. Med.* 19 35–42. 10.1038/nm.3028 23296015 PMC3998103

[B8] Bianco P. Kuznetsov S. A. Riminucci M. Gehron Robey P. (2006). Postnatal skeletal stem cells. *Methods Enzymol.* 419 117–148. 10.1016/S0076-6879(06)19006-0 17141054

[B9] Bianco P. Robey P. G. Simmons P. J. (2008). Mesenchymal stem cells: revisiting history, concepts, and assays. *Cell Stem Cell* 2 313–319. 10.1016/j.stem.2008.03.002 18397751 PMC2613570

[B10] Buechler M. B. Pradhan R. N. Krishnamurty A. T. Cox C. Calviello A. K. Wang A. W. (2021). Cross-tissue organization of the fibroblast lineage. *Nature* 593 575–579. 10.1038/s41586-021-03549-5 33981032

[B11] Buenrostro J. D. Wu B. Litzenburger U. M. Ruff D. Gonzales M. L. Snyder M. P. (2015). Single-cell chromatin accessibility reveals principles of regulatory variation. *Nature* 523 486–490. 10.1038/nature14590 26083756 PMC4685948

[B12] Campbell P. J. Green A. R. (2006). The myeloproliferative disorders. *N. Engl. J. Med.* 355 2452–2466. 10.1056/NEJMra063728 17151367

[B13] Cazzola M. Kralovics R. (2014). From Janus kinase 2 to calreticulin: the clinically relevant genomic landscape of myeloproliferative neoplasms. *Blood* 123 3714–3719. 10.1182/blood-2014-03-530865 24786775

[B14] Chagraoui H. Tulliez M. Smayra T. Komura E. Giraudier S. Yun T. (2003). Stimulation of osteoprotegerin production is responsible for osteosclerosis in mice overexpressing TPO. *Blood* 101 2983–2989. 10.1182/blood-2002-09-2839 12506018

[B15] Chan C. K. Seo E. Y. Chen J. Y. Lo D. McArdle A. Sinha R. (2015). Identification and specification of the mouse skeletal stem cell. *Cell* 160 285–298. 10.1016/j.cell.2014.12.002 25594184 PMC4297645

[B16] Chan C. K. F. Gulati G. S. Sinha R. Tompkins J. V. Lopez M. Carter A. C. (2018). Identification of the human skeletal stem cell. *Cell* 175 43–56.e21. 10.1016/j.cell.2018.07.029 30241615 PMC6400492

[B17] Ciurea S. O. Merchant D. Mahmud N. Ishii T. Zhao Y. Hu W. (2007). Pivotal contributions of megakaryocytes to the biology of idiopathic myelofibrosis. *Blood* 110 986–993. 10.1182/blood-2006-12-064626 17473062 PMC1924766

[B18] Costa L. A. Eiro N. Fraile M. Gonzalez L. O. Saa J. Garcia-Portabella P. (2021). Functional heterogeneity of mesenchymal stem cells from natural niches to culture conditions: implications for further clinical uses. *Cell. Mol. Life Sci.* 78 447–467. 10.1007/s00018-020-03600-0 32699947 PMC7375036

[B19] Crane G. M. Jeffery E. Morrison S. J. (2017). Adult haematopoietic stem cell niches. *Nat. Rev. Immunol.* 17 573–590. 10.1038/nri.2017.53 28604734

[B20] Crisan M. Yap S. Casteilla L. Chen C. W. Corselli M. Park T. S. (2008). A perivascular origin for mesenchymal stem cells in multiple human organs. *Cell Stem Cell* 3 301–313. 10.1016/j.stem.2008.07.003 18786417

[B21] Darnell M. O’Neil A. Mao A. Gu L. Rubin L. L. Mooney D. J. (2018). Material microenvironmental properties couple to induce distinct transcriptional programs in mammalian stem cells. *Proc. Natl. Acad. Sci. U.S.A.* 115 E8368–E8377. 10.1073/pnas.1802568115 30120125 PMC6130338

[B22] Decker M. Martinez-Morentin L. Wang G. Lee Y. Liu Q. Leslie J. (2017). Leptin-receptor-expressing bone marrow stromal cells are myofibroblasts in primary myelofibrosis. *Nat. Cell Biol.* 19 677–688. 10.1038/ncb3530 28481328 PMC5801040

[B23] Ding L. Morrison S. J. (2013). Haematopoietic stem cells and early lymphoid progenitors occupy distinct bone marrow niches. *Nature* 495 231–235. 10.1038/nature11885 23434755 PMC3600153

[B24] Ding L. Saunders T. L. Enikolopov G. Morrison S. J. (2012). Endothelial and perivascular cells maintain haematopoietic stem cells. *Nature* 481 457–462. 10.1038/nature10783 22281595 PMC3270376

[B25] Dominici M. Le Blanc K. Mueller I. Slaper-Cortenbach I. Marini F. Krause D. (2006). Minimal criteria for defining multipotent mesenchymal stromal cells. The International Society for Cellular Therapy position statement. *Cytotherapy* 8 315–317. 10.1080/14653240600855905 16923606

[B26] Doulatov S. Notta F. Laurenti E. Dick J. E. (2012). Hematopoiesis: a human perspective. *Cell Stem Cell* 10 120–136. 10.1016/j.stem.2012.01.006 22305562

[B27] El Agha E. Kramann R. Schneider R. K. Li X. Seeger W. Humphreys B. D. (2017). Mesenchymal stem cells in fibrotic disease. *Cell Stem Cell* 21 166–177. 10.1016/j.stem.2017.07.011 28777943

[B28] Forte D. Garcia-Fernandez M. Sanchez-Aguilera A. Stavropoulou V. Fielding C. Martin-Perez D. (2020). Bone marrow mesenchymal stem cells support acute myeloid leukemia bioenergetics and enhance antioxidant defense and escape from chemotherapy. *Cell Metab.* 32 829–843.e9. 10.1016/j.cmet.2020.09.001 32966766 PMC7658808

[B29] Friedenstein A. J. Deriglasova U. F. Kulagina N. N. Panasuk A. F. Rudakowa S. F. Luria E. A. (1974). Precursors for fibroblasts in different populations of hematopoietic cells as detected by the in vitro colony assay method. *Exp. Hematol.* 2 83–92. 4455512

[B30] Friedenstein A. J. Petrakova K. V. Kurolesova A. I. Frolova G. P. (1968). Heterotopic of bone marrow. Analysis of precursor cells for osteogenic and hematopoietic tissues. *Transplantation* 6 230–247. 5654088

[B31] Gomez D. Shankman L. S. Nguyen A. T. Owens G. K. (2013). Detection of histone modifications at specific gene loci in single cells in histological sections. *Nat. Methods* 10 171–177. 10.1038/nmeth.2332 23314172 PMC3560316

[B32] Graf T. Stadtfeld M. (2008). Heterogeneity of embryonic and adult stem cells. *Cell Stem Cell* 3 480–483. 10.1016/j.stem.2008.10.007 18983963

[B33] Gupta K. Levinsohn J. Linderman G. Chen D. Sun T. Y. Dong D. (2019). Single-cell analysis reveals a hair follicle dermal niche molecular differentiation trajectory that begins prior to morphogenesis. *Dev. Cell* 48 17–31.e6. 10.1016/j.devcel.2018.11.032 30595533 PMC6361530

[B34] Hara K. Nakagawa T. Enomoto H. Suzuki M. Yamamoto M. Simons B. D. (2014). Mouse spermatogenic stem cells continually interconvert between equipotent singly isolated and syncytial states. *Cell Stem Cell* 14 658–672. 10.1016/j.stem.2014.01.019 24792118 PMC4010676

[B35] Harada S. Mabuchi Y. Kohyama J. Shimojo D. Suzuki S. Kawamura Y. (2020). FZD5 regulates cellular senescence in human mesenchymal stem/stromal cells. *Stem Cells* 39 318–330. 10.1002/stem.3317 33338299 PMC7986096

[B36] Hayashi T. Ozaki H. Sasagawa Y. Umeda M. Danno H. Nikaido I. (2018). Single-cell full-length total RNA sequencing uncovers dynamics of recursive splicing and enhancer RNAs. *Nat. Commun.* 9:619. 10.1038/s41467-018-02866-0 29434199 PMC5809388

[B37] Isern J. Garcia-Garcia A. Martin A. M. Arranz L. Martin-Perez D. Torroja C. (2014). The neural crest is a source of mesenchymal stem cells with specialized hematopoietic stem cell niche function. *Elife* 3:e03696. 10.7554/eLife.03696 25255216 PMC4381911

[B38] Itkin T. Gur-Cohen S. Spencer J. A. Schajnovitz A. Ramasamy S. K. Kusumbe A. P. (2016). Distinct bone marrow blood vessels differentially regulate haematopoiesis. *Nature* 532 323–328. 10.1038/nature17624 27074509 PMC6450701

[B39] Jacobson R. J. Salo A. Fialkow P. J. (1978). Agnogenic myeloid metaplasia: a clonal proliferation of hematopoietic stem cells with secondary myelofibrosis. *Blood* 51 189–194. 620081

[B40] Kfoury Y. Scadden D. T. (2015). Mesenchymal cell contributions to the stem cell niche. *Cell Stem Cell* 16 239–253. 10.1016/j.stem.2015.02.019 25748931

[B41] Kim J. Kang J. W. Park J. H. Choi Y. Choi K. S. Park K. D. (2009). Biological characterization of long-term cultured human mesenchymal stem cells. *Arch. Pharm. Res.* 32 117–126. 10.1007/s12272-009-1125-1 19183884

[B42] Kunisaki Y. Bruns I. Scheiermann C. Ahmed J. Pinho S. Zhang D. (2013). Arteriolar niches maintain haematopoietic stem cell quiescence. *Nature* 502 637–643. 10.1038/nature12612 24107994 PMC3821873

[B43] Kurenkova A. D. Medvedeva E. V. Newton P. T. Chagin A. S. (2020). Niches for skeletal stem cells of mesenchymal origin. *Front. Cell Dev. Biol.* 8:592. 10.3389/fcell.2020.00592 32754592 PMC7366157

[B44] Lataillade J. J. Pierre-Louis O. Hasselbalch H. C. Uzan G. Jasmin C. Martyre M. C. (2008). Does primary myelofibrosis involve a defective stem cell niche? From concept to evidence. *Blood* 112 3026–3035. 10.1182/blood-2008-06-158386 18669872

[B45] Lee H. Shimotakahara R. Fukada A. Shinbashi S. Ogata S. (2019). Impact of differences in clinical training methoent of nursing students: a text mining analysis study. *Heliyon* 5:e01285. 10.1016/j.heliyon.2019.e01285 30923759 PMC6423991

[B46] Leimkuhler N. B. Gleitz H. F. E. Ronghui L. Snoeren I. A. M. Fuchs S. N. R. Nagai J. S. (2020). Heterogeneous bone-marrow stromal progenitors drive myelofibrosis via a druggable alarmin axis. *Cell Stem Cell* 28 637–652.e8. 10.1016/j.stem.2020.11.004 33301706 PMC8024900

[B47] Litvinukova M. Talavera-Lopez C. Maatz H. Reichart D. Worth C. L. Lindberg E. L. (2020). Cells of the adult human heart. *Nature* 588 466–472. 10.1038/s41586-020-2797-4 32971526 PMC7681775

[B48] Mabuchi Y. Houlihan D. D. Akazawa C. Okano H. Matsuzaki Y. (2013a). Prospective isolation of murine and human bone marrow mesenchymal stem cells based on surface markers. *Stem Cells Int.* 2013:507301. 10.1155/2013/507301 23766770 PMC3673454

[B49] Mabuchi Y. Matsuzaki Y. (2016). Prospective isolation of resident adult human mesenchymal stem cell population from multiple organs. *Int. J. Hematol.* 103 138–144. 10.1007/s12185-015-1921-y 26676805

[B50] Mabuchi Y. Morikawa S. Harada S. Niibe K. Suzuki S. Renault-Mihara F. (2013b). LNGFR(+)THY-1(+)VCAM-1(hi+) cells reveal functionally distinct subpopulations in mesenchymal stem cells. *Stem Cell Rep.* 1 152–165. 10.1016/j.stemcr.2013.06.001 24052950 PMC3757748

[B51] Macosko E. Z. Basu A. Satija R. Nemesh J. Shekhar K. Goldman M. (2015). Highly parallel genome-wide expression profiling of individual cells using nanoliter droplets. *Cell* 161 1202–1214. 10.1016/j.cell.2015.05.002 26000488 PMC4481139

[B52] Mendez-Ferrer S. Michurina T. V. Ferraro F. Mazloom A. R. Macarthur B. D. Lira S. A. (2010). Mesenchymal and haematopoietic stem cells form a unique bone marrow niche. *Nature* 466 829–834. 10.1038/nature09262 20703299 PMC3146551

[B53] Mizoguchi T. Pinho S. Ahmed J. Kunisaki Y. Hanoun M. Mendelson A. (2014). Osterix marks distinct waves of primitive and definitive stromal progenitors during bone marrow development. *Dev. Cell* 29 340–349. 10.1016/j.devcel.2014.03.013 24823377 PMC4051418

[B54] Morikawa S. Mabuchi Y. Kubota Y. Nagai Y. Niibe K. Hiratsu E. (2009). Prospective identification, isolation, and systemic transplantation of multipotent mesenchymal stem cells in murine bone marrow. *J. Exp. Med.* 206 2483–2496. 10.1084/jem.20091046 19841085 PMC2768869

[B55] Nagoshi N. Shibata S. Kubota Y. Nakamura M. Nagai Y. Satoh E. (2008). Ontogeny and multipotency of neural crest-derived stem cells in mouse bone marrow, dorsal root ganglia, and whisker pad. *Cell Stem Cell* 2 392–403. 10.1016/j.stem.2008.03.005 18397758

[B56] Nolta J. A. Galipeau J. Phinney D. G. (2020). Improving mesenchymal stem/stromal cell potency and survival: proceedings from the International Society of Cell Therapy (ISCT) MSC preconference held in May 2018, Palais des Congres de Montreal, Organized by the ISCT MSC Scientific Committee. *Cytotherapy* 22 123–126. 10.1016/j.jcyt.2020.01.004 32067856

[B57] Notta F. Doulatov S. Laurenti E. Poeppl A. Jurisica I. Dick J. E. (2011). Isolation of single human hematopoietic stem cells capable of long-term multilineage engraftment. *Science* 333 218–221. 10.1126/science.1201219 21737740

[B58] Ogata Y. Mabuchi Y. Yoshida M. Suto E. G. Suzuki N. Muneta T. (2015). Purified human synovium mesenchymal stem cells as a good resource for cartilage regeneration. *PLoS One* 10:e0129096. 10.1371/journal.pone.0129096 26053045 PMC4459808

[B59] Oguro H. Ding L. Morrison S. J. (2013). SLAM family markers resolve functionally distinct subpopulations of hematopoietic stem cells and multipotent progenitors. *Cell Stem Cell* 13 102–116. 10.1016/j.stem.2013.05.014 23827712 PMC3736853

[B60] Osawa M. Hanada K. Hamada H. Nakauchi H. (1996). Long-term lymphohematopoietic reconstitution by a single CD34-low/negative hematopoietic stem cell. *Science* 273 242–245. 10.1126/science.273.5272.242 8662508

[B61] Ozono Y. Shide K. Kameda T. Kamiunten A. Tahira Y. Sekine M. (2020). Neoplastic fibrocytes play an essential role in bone marrow fibrosis in Jak2V617F-induced primary myelofibrosis mice. *Leukemia* 35 454–467. 10.1038/s41375-020-0880-3 32472085 PMC7862060

[B62] Peterson V. M. Zhang K. X. Kumar N. Wong J. Li L. Wilson D. C. (2017). Multiplexed quantification of proteins and transcripts in single cells. *Nat. Biotechnol.* 35 936–939. 10.1038/nbt.3973 28854175

[B63] Pittenger M. F. Mackay A. M. Beck S. C. Jaiswal R. K. Douglas R. Mosca J. D. (1999). Multilineage potential of adult human mesenchymal stem cells. *Science* 284 143–147. 10.1126/science.284.5411.143 10102814

[B64] Raj A. van den Bogaard P. Rifkin S. A. van Oudenaarden A. Tyagi S. (2008). Imaging individual mRNA molecules using multiple singly labeled probes. *Nat. Methods* 5 877–879. 10.1038/nmeth.1253 18806792 PMC3126653

[B65] Raj B. Wagner D. E. McKenna A. Pandey S. Klein A. M. Shendure J. (2018). Simultaneous single-cell profiling of lineages and cell types in the vertebrate brain. *Nat. Biotechnol.* 36 442–450. 10.1038/nbt.4103 29608178 PMC5938111

[B66] Ren J. Wang H. Tran K. Civini S. Jin P. Castiello L. (2015). Human bone marrow stromal cell confluence: effects on cell characteristics and methods of assessment. *Cytotherapy* 17 897–911. 10.1016/j.jcyt.2015.03.607 25882666 PMC4461557

[B67] Rennerfeldt D. A. Van Vliet K. J. (2016). Concise review: when colonies are not clones: evidence and implications of intracolony heterogeneity in mesenchymal stem cells. *Stem Cells* 34 1135–1141. 10.1002/stem.2296 26840390

[B68] Rodda L. B. Lu E. Bennett M. L. Sokol C. L. Wang X. Luther S. A. (2018). Single-cell RNA sequencing of lymph node stromal cells reveals niche-associated heterogeneity. *Immunity* 48 1014–1028.e6. 10.1016/j.immuni.2018.04.006 29752062 PMC5971117

[B69] Roson-Burgo B. Sanchez-Guijo F. Del Canizo C. De Las Rivas J. (2014). Transcriptomic portrait of human mesenchymal stromal/stem cells isolated from bone marrow and placenta. *BMC Genomics* 15:910. 10.1186/1471-2164-15-910 25326687 PMC4287589

[B70] Roson-Burgo B. Sanchez-Guijo F. Del Canizo C. De Las Rivas J. (2016). Insights into the human mesenchymal stromal/stem cell identity through integrative transcriptomic profiling. *BMC Genomics* 17:944. 10.1186/s12864-016-3230-0 27871224 PMC5117530

[B71] Sacchetti B. Funari A. Michienzi S. Di Cesare S. Piersanti S. Saggio I. (2007). Self-renewing osteoprogenitors in bone marrow sinusoids can organize a hematopoietic microenvironment. *Cell* 131 324–336. 10.1016/j.cell.2007.08.025 17956733

[B72] Sacchetti B. Funari A. Remoli C. Giannicola G. Kogler G. Liedtke S. (2016). No identical “Mesenchymal Stem Cells” at different times and sites: human committed progenitors of distinct origin and differentiation potential are incorporated as adventitial cells in microvessels. *Stem Cell Rep.* 6 897–913. 10.1016/j.stemcr.2016.05.011 27304917 PMC4912436

[B73] Sanchez-Luis E. Joaquin-Garcia A. Campos-Laborie F. J. Sanchez-Guijo F. Rivas J. L. (2020). Deciphering master gene regulators and associated networks of human mesenchymal stromal cells. *Biomolecules* 10:557. 10.3390/biom10040557 32260546 PMC7226324

[B74] Sasagawa Y. Nikaido I. Hayashi T. Danno H. Uno K. D. Imai T. (2013). Quartz-Seq: a highly reproducible and sensitive single-cell RNA sequencing method, reveals non-genetic gene-expression heterogeneity. *Genome Biol.* 14:3097. 10.1186/gb-2013-14-4-r31 23594475 PMC4054835

[B75] Sato T. Laver J. H. Ogawa M. (1999). Reversible expression of CD34 by murine hematopoietic stem cells. *Blood* 94 2548–2554. 10515856

[B76] Schepers K. Campbell T. B. Passegue E. (2015). Normal and leukemic stem cell niches: insights and therapeutic opportunities. *Cell Stem Cell* 16 254–267. 10.1016/j.stem.2015.02.014 25748932 PMC4391962

[B77] Schmitt A. Drouin A. Masse J. M. Guichard J. Shagraoui H. Cramer E. M. (2002). Polymorphonuclear neutrophil and megakaryocyte mutual involvement in myelofibrosis pathogenesis. *Leuk. Lymphoma* 43 719–724. 10.1080/10428190290016809 12153156

[B78] Schneider R. K. Mullally A. Dugourd A. Peisker F. Hoogenboezem R. Van Strien P. M. H. (2017). Gli1(+) mesenchymal stromal cells are a key driver of bone marrow fibrosis and an important cellular therapeutic target. *Cell Stem Cell* 20 785–800.e8. 10.1016/j.stem.2017.03.008 28457748 PMC6485654

[B79] Schneider R. K. Schenone M. Ferreira M. V. Kramann R. Joyce C. E. Hartigan C. (2016). Rps14 haploinsufficiency causes a block in erythroid differentiation mediated by S100A8 and S100A9. *Nat. Med.* 22, 288–297. 10.1038/nm.4047 26878232 PMC4870050

[B80] Schofield R. (1978). The relationship between the spleen colony-forming cell and the haemopoietic stem cell. *Blood Cells* 4, 7–25. 747780

[B81] Sena I. F. G. Borges I. T. Lousado L. Azevedo P. O. Andreotti J. P. Almeida V. M. (2017). LepR+ cells dispute hegemony with Gli1+ cells in bone marrow fibrosis. *Cell Cycle* 16 2018–2022. 10.1080/15384101.2017.1367072 28976809 PMC5731410

[B82] Severe N. Karabacak N. M. Gustafsson K. Baryawno N. Courties G. Kfoury Y. (2019). Stress-induced changes in bone marrow stromal cell populations revealed through single-cell protein expression mapping. *Cell Stem Cell* 25, 570–583. 10.1016/j.stem.2019.06.003 31279774 PMC6778015

[B83] Smallwood S. A. Lee H. J. Angermueller C. Krueger F. Saadeh H. Peat J. (2014). Single-cell genome-wide bisulfite sequencing for assessing epigenetic heterogeneity. *Nat. Methods* 11 817–820. 10.1038/nmeth.3035 25042786 PMC4117646

[B84] Spanjaard B. Hu B. Mitic N. Olivares-Chauvet P. Janjuha S. Ninov N. (2018). Simultaneous lineage tracing and cell-type identification using CRISPR-Cas9-induced genetic scars. *Nat. Biotechnol.* 36 469–473. 10.1038/nbt.4124 29644996 PMC5942543

[B85] Stagg J. Pommey S. Eliopoulos N. Galipeau J. (2006). Interferon-gamma-stimulated marrow stromal cells: a new type of nonhematopoietic antigen-presenting cell. *Blood* 107 2570–2577. 10.1182/blood-2005-07-2793 16293599

[B86] Stoeckius M. Hafemeister C. Stephenson W. Houck-Loomis B. Chattopadhyay P. K. Swerdlow H. (2017). Simultaneous epitope and transcriptome measurement in single cells. *Nat. Methods* 14 865–868. 10.1038/nmeth.4380 28759029 PMC5669064

[B87] Stuart T. Satija R. (2019). Integrative single-cell analysis. *Nat. Rev. Genet.* 20 257–272. 10.1038/s41576-019-0093-7 30696980

[B88] Sugiyama T. Kohara H. Noda M. Nagasawa T. (2006). Maintenance of the hematopoietic stem cell pool by CXCL12-CXCR4 chemokine signaling in bone marrow stromal cell niches. *Immunity* 25 977–988. 10.1016/j.immuni.2006.10.016 17174120

[B89] Sun W. Dong H. Balaz M. Slyper M. Drokhlyansky E. Colleluori G. (2020). snRNA-seq reveals a subpopulation of adipocytes that regulates thermogenesis. *Nature* 587 98–102. 10.1038/s41586-020-2856-x 33116305

[B90] Suto E. G. Mabuchi Y. Toyota S. Taguchi M. Naraoka Y. Itakura N. (2020). Advantage of fat-derived CD73 positive cells from multiple human tissues, prospective isolated mesenchymal stromal cells. *Sci. Rep.* 10:15073. 10.1038/s41598-020-72012-8 32934322 PMC7493914

[B91] Tabarroki A. Tiu R. V. (2014). Molecular genetics of myelofibrosis and its associated disease phenotypes. *Transl. Med. UniSa* 8 53–64. 24778998 PMC4000463

[B92] Takashima Y. Era T. Nakao K. Kondo S. Kasuga M. Smith A. G. (2007). Neuroepithelial cells supply an initial transient wave of MSC differentiation. *Cell* 129 1377–1388. 10.1016/j.cell.2007.04.028 17604725

[B93] Tanay A. Regev A. (2017). Scaling single-cell genomics from phenomenology to mechanism. *Nature* 541 331–338. 10.1038/nature21350 28102262 PMC5438464

[B94] Tikhonova A. N. Dolgalev I. Hu H. Sivaraj K. K. Hoxha E. Cuesta-Dominguez A. (2019). The bone marrow microenvironment at single-cell resolution. *Nature* 569 222–228. 10.1038/s41586-019-1104-8 30971824 PMC6607432

[B95] Tomuleasa C. Selicean S. Gafencu G. Petrushev B. Pop L. Berce C. (2018). Fibroblast dynamics as an in vitro screening platform for anti-fibrotic drugs in primary myelofibrosis. *J. Cell. Physiol.* 233 422–433. 10.1002/jcp.25902 28294327

[B96] Travaglini K. J. Nabhan A. N. Penland L. Sinha R. Gillich A. Sit R. V. (2020). A molecular cell atlas of the human lung from single-cell RNA sequencing. *Nature* 587 619–625. 10.1038/s41586-020-2922-4 33208946 PMC7704697

[B97] Turinetto V. Vitale E. Giachino C. (2016). Senescence in human mesenchymal stem cells: functional changes and implications in stem cell-based therapy. *Int. J. Mol. Sci.* 17:1164. 10.3390/ijms17071164 27447618 PMC4964536

[B98] Vitak S. A. Torkenczy K. A. Rosenkrantz J. L. Fields A. J. Christiansen L. Wong M. H. (2017). Sequencing thousands of single-cell genomes with combinatorial indexing. *Nat. Methods* 14 302–308. 10.1038/nmeth.4154 28135258 PMC5908213

[B99] Watanabe H. Okuda R. Hagino H. (2018). Core values in nursing care based on the experiences of nurses engaged in neonatal nursing: a text-mining approach for analyzing reflection records. *Yonago Acta Med.* 61 40–48. 29599621 10.33160/yam.2018.03.006 PMC5871725

[B100] Watt F. M. Hogan B. L. (2000). Out of Eden: stem cells and their niches. *Science* 287 1427–1430. 10.1126/science.287.5457.1427 10688781

[B101] Wolock S. L. Krishnan I. Tenen D. E. Matkins V. Camacho V. Patel S. (2019). Mapping distinct bone marrow niche populations and their differentiation paths. *Cell Rep.* 28 302–311.e5. 10.1016/j.celrep.2019.06.031 31291568 PMC6684313

[B102] Worthley D. L. Churchill M. Compton J. T. Tailor Y. Rao M. Si Y. (2015). Gremlin 1 identifies a skeletal stem cell with bone, cartilage, and reticular stromal potential. *Cell* 160 269–284. 10.1016/j.cell.2014.11.042 25594183 PMC4436082

[B103] Yamazaki S. Ema H. Karlsson G. Yamaguchi T. Miyoshi H. Shioda S. (2011). Nonmyelinating Schwann cells maintain hematopoietic stem cell hibernation in the bone marrow niche. *Cell* 147 1146–1158. 10.1016/j.cell.2011.09.053 22118468

[B104] Yasui T. Mabuchi Y. Toriumi H. Ebine T. Niibe K. Houlihan D. D. (2016). Purified human dental pulp stem cells promote osteogenic regeneration. *J. Dent. Res.* 95 206–214. 10.1177/0022034515610748 26494655

[B105] Yoshida S. (2018). Open niche regulation of mouse spermatogenic stem cells. *Dev. Growth Differ.* 60 542–552. 10.1111/dgd.12574 30443901 PMC11520966

[B106] Yue R. Zhou B. O. Shimada I. S. Zhao Z. Morrison S. J. (2016). Leptin receptor promotes adipogenesis and reduces osteogenesis by regulating mesenchymal stromal cells in adult bone marrow. *Cell Stem Cell* 18 782–796. 10.1016/j.stem.2016.02.015 27053299

[B107] Zetterberg E. Verrucci M. Martelli F. Zingariello M. Sancillo L. D’Amore E. (2014). Abnormal P-selectin localization during megakaryocyte development determines thrombosis in the gata1low model of myelofibrosis. *Platelets* 25 539–547. 10.3109/09537104.2013.840720 24176039 PMC4045657

[B108] Zhong L. Yao L. Tower R. J. Wei Y. Miao Z. Park J. (2020). Single cell transcriptomics identifies a unique adipose lineage cell population that regulates bone marrow environment. *Elife* 9:e54695. 10.7554/eLife.54695 32286228 PMC7220380

[B109] Zhou B. O. Yu H. Yue R. Zhao Z. Rios J. J. Naveiras O. (2017). Bone marrow adipocytes promote the regeneration of stem cells and haematopoiesis by secreting SCF. *Nat. Cell Biol.* 19 891–903. 10.1038/ncb3570 28714970 PMC5536858

[B110] Zhou B. O. Yue R. Murphy M. M. Peyer J. G. Morrison S. J. (2014). Leptin-receptor-expressing mesenchymal stromal cells represent the main source of bone formed by adult bone marrow. *Cell Stem Cell* 15 154–168. 10.1016/j.stem.2014.06.008 24953181 PMC4127103

